# Natural-Product-Derived Adjunctive Treatments to Conventional Therapy and Their Immunoregulatory Activities in Triple-Negative Breast Cancer

**DOI:** 10.3390/molecules28155804

**Published:** 2023-08-01

**Authors:** Lea Ling-Yu Kan, Ben Chung-Lap Chan, Ping-Chung Leung, Chun-Kwok Wong

**Affiliations:** 1 Institute of Chinese Medicine, The Chinese University of Hong Kong, Hong Kong, China; lea_kan@link.cuhk.edu.hk (L.L.-Y.K.); benchan99@cuhk.edu.hk (B.C.-L.C.); pingcleung@cuhk.edu.hk (P.-C.L.); 2 State Key Laboratory of Research on Bioactivities and Clinical Applications of Medicinal Plants, The Chinese University of Hong Kong, Hong Kong, China; 3 Department of Chemical Pathology, The Chinese University of Hong Kong, Hong Kong, China; 4 Li Dak Sum Yip Yio Chin R & D Centre for Chinese Medicine, The Chinese University of Hong Kong, Hong Kong, China

**Keywords:** adjunctive immunotherapy, tumor microenvironment, natural products, cancer

## Abstract

Triple-negative breast cancer (TNBC) is an invasive and persistent subtype of breast cancer that is likely to be resistant to conventional treatments. The rise in immunotherapy has created new modalities to treat cancer, but due to high costs and unreliable efficacy, adjunctive and complementary treatments have sparked interest in enhancing the efficacy of currently available treatments. Natural products, which are bioactive compounds derived from natural sources, have historically been used to treat or ameliorate inflammatory diseases and symptoms. As TNBC patients have shown little to no response to immunotherapy, the potential of natural products as candidates for adjuvant immunotherapy is being explored, as well as their immunomodulatory effects on cancer. Due to the complexity of TNBC and the ever-changing tumor microenvironment, there are challenges in determining the feasibility of using natural products to enhance the efficacy or counteract the toxicity of conventional treatments. In view of technological advances in molecular docking, pharmaceutical networking, and new drug delivery systems, natural products show promise as potential candidates in adjunctive therapy. In this article, we summarize the mechanisms of action of selected natural-product-based bioactive compounds and analyze their roles and applications in combination treatments and immune regulation.

## 1. Introduction

Cancer is a chronic inflammatory disease that is well adapted to proliferating unchecked and evading detection by the immune system. Breast cancer dominates in global prevalence and incidence out of all cancers, with over 2.26 million cases reported in 2020 alone [1]. Currently, an estimated 83% of those diagnosed with breast cancer are over 50 years of age; therefore, with an increasing proportion of aging populations in high- and middle-income countries, the incidence of breast cancer is likely to exceed 3 million cases per year by 2040 [2]. In the United States, for example, 13% of women are at risk of developing invasive breast cancer in their lifetime, and 2.5% will die from it [3]. Hereditary factors such as deleterious mutations in breast cancer susceptibility proteins type 1 (BRCA1) and type 2 (BRCA2) account for up to 10% of breast cancer cases; therefore, most cases are caused by extrinsic risk factors [4]. Modifiable risk factors include physical inactivity, obesity, and alcohol consumption, while non-modifiable risk factors include mammographic density, reproductive history, hormonal changes, and childhood exposure to ionizing radiation [5,6].

Breast cancer is classified into four main subtypes, which are distinguished by their molecular phenotype: estrogen receptor (ER)-positive, progesterone receptor (PR)-positive, human epidermal growth factor 2 receptor (HER)-positive, or triple-negative breast cancer (TNBC; the absence of all receptors) [7]. TNBC is one of the most invasive and aggressive forms of breast cancer, accounting for approximately 15–20% of all breast cancers [8]. It is also associated with a poorer prognosis and higher risk of recurrence, with a 5-year relative survival rate of 77.6% compared with 90.5% for non-TNBC in the United States [9]. Breast cancer subtype and malignancy are the key determinants of patient prognosis and treatment of choice. Most breast cancer patients are treated by surgical removal of the primary tumor before receiving adjuvant therapy, a combination of chemotherapy, radiotherapy, and/or endocrine therapy [10]. Neoadjuvant therapy, treatment prior to surgical removal of the tumor, may also be used in selected candidates who are eligible according to the recommended guidelines and medical practitioner approval [11,12]. Although adjuvant and neoadjuvant treatments are equally effective in prolonging survival rates, drug delivery is highly non-specific, causing adverse effects in patients and compromising their well-being. This has led to the development of endocrine therapeutic drugs such as tamoxifen, which specifically targets ER-positive cancers [13,14]. However, the duration of treatment can be up to 10 years to order to improve survival rates and prevent cancer recurrence [15].

In the 1800s, Busch, Fehleisen, and William Coley were among the first to observe that tumor regression could be achieved by harnessing the immune response. Coley was the first to demonstrate the reversal of malignancy by injecting sarcoma patients with heat-inactivated *Streptococcus pyogenes* and *Serratia marcescens*, bacterial species that cause erysipelas [16,17]. By 2014, the development of immunotherapy drugs had revolutionized modern cancer treatment by targeting and eliminating cancer cells by directly inducing immune-mediated cell death. Three main immunotherapeutic approaches are now available: adoptive T-cell therapy, immune checkpoint blockade, and cancer vaccines [18]. Genome modification by clustered regularly interspaced short palindromic repeats (CRISPR)-associated protein 9 (CRISPR-Cas9) technology is now being used to support adoptive T cell therapy to alter cancer or immune cell function. This technology can be used for ex vivo knockout of the immune checkpoint proteins from tumor-infiltrating T cells, knockout of endogenous T cell receptors (TCRs) followed by knock-in of cancer antigen-specific TCRs, and knock-in of chimeric-antigen receptors into T cells [19,20,21]. This is a promising approach for cancer treatment in immunogenic tumors to minimize the systemic destruction of healthy cells caused by chemo- or radiotherapy-induced toxicity. However, clinical trials have shown that only a small fraction of patients respond to treatment, while the majority have no response. This represents the establishment of primary, adaptive, or acquired resistance [22]. In addition, tumor heterogeneity, immunosuppression within the tumor microenvironment, drug resistance, gut microbiota, and tumor mutational burden (TMB) can impede drug delivery. Drug-induced T-cell activation can cause non-specific tissue damage that manifests as immune-related adverse events and, if severe, can lead to the development of various autoimmune diseases such as inflammatory rheumatoid arthritis [23,24,25]. Patients who show no response or partial response to immunotherapy may develop resistance, leading to an increased likelihood of cancer recurrence through the formation of cell-in-cell structures [26]. Dormant cancer cells may undergo further mutations before initiating cancer recurrence at distant or loco-regional sites. Emerging evidence suggests that in breast cancer subtypes, discordance between the primary tumor site and the metastatic site may occur during tumor recurrence, such as the alteration or loss of hormone receptor expression. This can exacerbate cancer status and treatment strategies must adapt to these changes to maximize patient survival [27,28].

In recent years, complementary and alternative therapies have been gaining worldwide recognition for their application in conventional cancer treatment. Natural products are broadly defined as bioactive extracts, metabolites, or derivatives of natural origin, such as plants, animals, fungi, and microorganisms [29]. For example, medicinal herbs used in traditional Chinese medicine (TCM) are known to be used in adjuvant therapy against chronic inflammatory diseases and provide immunological support to facilitate patient recovery [30]. Studies have reported that herbal medicine is successful in improving the well-being of recovering cancer patients, as evidenced by the reduction of adverse effects of conventional treatments, stimulation of the anti-cancer immune response, and reversal of drug resistance [31]. Although herbal medicine mainly boosts systemic immunity including innate and adaptive immunity, its derivatives provide important aspects of drugs, precise immunological pathways, and the efficacy of chemotherapy, radiotherapy, endocrine therapy, and immunotherapy.

This review discusses the potential adjuvant use of natural products in the treatment of TNBC with immunotherapy, their mechanisms of action in immunomodulation, and their influence on the tumor microenvironment (TME). The synergistic anti-tumor mechanisms between immunotherapeutic drugs and immune-enhancing natural products, as well as their compatibility in clinical application and combination therapy, will be examined.

## 2. The Immune System and TNBC

### 2.1. Cancer Evasion of Host Immune Surveillance

Hanahan, D. has described more than 15 hallmarks of cancer cell survival adaptations, one of the key features being the prevention of immune-mediated destruction [32]. The cancer-immunity cycle is representative of the sustained immunosuppression of the immune response that allows tumor cells to evade immunosurveillance, thereby providing optimal conditions for uncontrolled proliferation [33]. First, neoantigens released from dead tumor cells are captured by nearby dendritic cells (DCs), which then migrate to the lymph nodes to prime naïve T cells. After priming and activation, CD8^+^ cytotoxic T (Tc) cells travel through the bloodstream to the tumor site. At the tumor site, tumor cells inactivate the CD8^+^ Tc cell activities by the binding of immune checkpoint ligands, allowing for the further proliferation and invasion of cancer cells [33,34].

There have been conflicting theories regarding the regulation between cancer development and immune surveillance. Paul Ehrlich first proposed the concept of the spontaneous elimination of transformed cells by immune cells, which formed the basis of the immunosurveillance hypothesis [35,36]. Thomas Lewis developed the early immunosurveillance theory, which was later refined by Frank McFarlane Burnet. It was hypothesized that the immune system would readily eradicate neoplastic cells, similar to homograft rejection, provided that neoantigens were present and recognized by the immune system [37,38].

This was refuted in later years by contradictory observations that tumors continue to form despite a fully competent immune system. The recognition of neoantigens and discrimination between self and non-self-antigens is largely regulated by the NKG2D receptors present on natural killer (NK) cells. NKG2D ligands are upregulated in transformed or virus-infected cells in response to the DNA damage response, a process common in breast cancer [39]. NKG2D ligands on cancer cells activate NK cells and other T cells to mediate the cell death mechanism. However, cancer cells can shed NKG2D ligands into a soluble form to escape detection before undergoing immunoediting and immunosubversion [40,41]. Immunoediting is defined as the process by which tumors evolve mechanisms to evade immune-mediated destruction. The principle is described in three stages: elimination, equilibrium, and escape [42]. The elimination phase refers to the complete clearance of tumor cells by immune cells upon the detection of neoantigens expressed on the cell surface. In the equilibrium phase, a few tumor cells that have survived or circumvented immunosurveillance may mutate to further enhance immune resistance. As a result, the overall cytotoxic immune response stalls, leading to tumor dormancy. The escape phase refers to the continued growth of immune-resistant tumor cells with little or no inhibition by a competent immune system, requiring the evasion of both adaptive and innate immune responses [43,44]. One study highlighted the paradoxical role of the intrinsic expression of interferon (IFN)-γ and functional lymphocytes. Both work in tandem to protect against tumorigenesis while promoting the immunoselection of tumor cells. This is also a key factor in dictating the extent and outcome of immunoediting [45].

Immunoediting involves a combination of intrinsic and extrinsic mechanisms to escape immune detection. Genomic profiling and signal transduction analyses have reported that mutations or loss of the RB1, TP53, and PTEN genes and activation of the PI3K and MEK pathways are found in many TNBC cases. Somatic alterations in these genes are prevalent features of metastasis in most solid tumors [46,47,48]. TNBC can also lead to mutations of caspase 8, allowing for the survival of tumor cells and preventing destruction by CD8^+^ cytotoxic T (Tc) cells [49]. Another genomic profiling study has shown that the immune evasion mechanisms of TNBC are adapted according to the tumor landscape as the phenotypes vary from one individual to another. The main mechanisms include the recruitment and trafficking of immune cells, activation or inactivation of immune cells, and expression of immune checkpoint proteins [50].

### 2.2. The Tumor Microenvironment and Immunogenicity

Tumor immunogenicity is defined as the tendency of the tumor to elicit an adaptive immune response from the host and it is primarily determined by antigenicity and adjuvanticity. Antigenicity represents the activation of the immune response via antigen binding, whereas adjuvanticity is the ability to potentiate the immune response against cancer cells [51,52]. Tumor immunogenicity can be measured by the TMB, the number of somatic mutations present in tumor cells. Tumor genetic profiling studies have shown that TMB in TNBC correlates with tumor-infiltrating lymphocytes (TILs), and both parameters are indicators of positive responses to immune checkpoint inhibitor (ICI) treatment and patient prognosis [53,54,55]. Cancers with a high TMB would exhibit a higher number of neoantigens and thus be detected more effectively by the immune system. Several factors influence TMB, including the genetic composition of cancer cells, the TME, and the immune status of the patient [56].

The TME is an integrated network consisting of the tumor stroma, vasculature, TILs, and other structural cells [57]. Tumor-infiltrating lymphocytes generally represent the CD8^+^ Tc cell population, an important measure of tumor immunogenicity. The TME can be categorized according to the distribution and degree of TIL infiltration: high TIL infiltration is described as immune-inflamed or immunogenically “hot”, TILs present at the tumor periphery without infiltration are described as immune-excluded or immunologically “cold”, and low or no TIL infiltration is described as immune-desert or immunologically “cold” [58,59]. Immunogenically “hot” tumors are more responsive to immunotherapy. TNBC patients with a high ratio of CD8^+^ and CD4^+^ T cells infiltrated in their tumors suggest a better prognosis and higher sensitivity to immunotherapy [60,61].

All leukocytes, except natural killer (NK) cells, can switch their phenotypic expression and functional properties to pro-tumorigenic, anti-tumorigenic, or non-reactive tendencies within the TME [62]. The major immunosuppressive cells in the TME of TNBC include myeloid-derived suppressor cells (MDSCs), tumor-associated macrophages (TAMs) with an M2 phenotype, tumor-associated neutrophils (TANs) with an N2 phenotype, regulatory T (Treg) cells, and CD4^+^ helper T (Th) cells. Immunostimulatory cells include TAMs and TANs with an M1 or N1 phenotype, CD8^+^ cytotoxic T cells, and NK cells [63,64]. During TNBC progression, the recruitment of immunosuppressive cells deactivates T cells through the release of the immunosuppressive cytokines transforming growth factor beta (TGF-β) and interleukin (IL)-10. Tumor-derived exosomes also contribute to T-cell exhaustion, which promotes metastasis and invasion [65,66].

### 2.3. Systemic Inflammation

Inflammation exists as acute or chronic inflammation, with different outcomes depending on stimuli and immunological status. In the context of non-pathogenic cancer, low-grade chronic inflammation persists at all stages of cancer development, from initiation to development and metastasis [67]. However, the causal link between carcinogenesis and inflammation is not fully understood and is often contradictory. For example, some chronic low-grade inflammatory diseases such as obesity and inflammatory bowel disease (IBD) predispose patients to developing breast and gastrointestinal cancers, respectively, while other diseases, such as rheumatoid arthritis, do not [62,68]. Only a small percentage of cancer patients have an intrinsically higher risk of developing cancer due to germline mutations, while most cancer patients are already in a pro-inflammatory state before they develop the disease due to somatic mutations [62].

Harold Dvorak, a renowned pathologist, described tumors as wounds that never heal. This comparison highlights the similarity between tumors and unresolved inflammation in wounds [69]. Acute inflammation occurs during tissue injury and infection, where cell damage and death trigger the production of damage-associated molecular patterns (DAMPs). Upon recognition by neighboring cells, pro-inflammatory chemicals signal the recruitment of immune cells to neutralize inflammation. Specialized pro-resolving mediators (SPMs) program the clearance of inflammatory mediators and inhibit the tissue-damaging response of neutrophils, followed by the proliferative cue to restore tissue homeostasis and epithelial structure [70,71,72]. However, incomplete elimination of pathogens or prolonged and unresolved acute inflammation leads to chronic inflammation development and the migration of immunosuppressive cells to the site of infection [73].

Cancer-associated inflammation (CAI) is characteristic of all stages of tumor development, from pre-tumor stages to cancer-therapy-induced inflammation [70]. It has been shown that CAI is regulated by intrinsic and extrinsic pathways, the intrinsic being oncogene activation or the silencing of tumor suppressor genes and the extrinsic being factors beyond the tumor cell that promote cancer, such as cytokine and chemokine production [73]. For example, the loss of the p53 gene in cancer cells drives the production of WNT ligands to release IL-1β, promoting metastasis and inflammation [71,74,75]. The net effects of pro- and anti-inflammatory processes in the TME determine tumor outcomes [76]. The proliferative activities of cancer cells are activated by the NF-κB signaling pathway and its downstream components, STAT 3 and IL-6. These transcription factors are pro-inflammatory in nature and are derived from innate immunity, shaping the phenotypes and functions of cells within the TME.

The adaptive immune response in cancer is an important regulator of pro- and anti-tumorigenic inflammation, in which T lymphocytes are actively involved [77]. CD8^+^ Tc cells participate in acute inflammation, whereas CD4^+^ helper T (Th) cells participate in acute or chronic inflammation, depending on their Th1 and Th2 lineages. Th1 cells release the proinflammatory cytokines IL-2 and IFN-γ, contributing to the phenotypic drift of M1 macrophages, and activate Tc cells to initiate tumor cell killing. Th2 cells release the immunosuppressive cytokines IL-4 and IL-10 to promote M2 macrophages [78]. CD4^+^ CD25^+^ FoxP3^+^ Treg cells are immunosuppressive cells that promote tumor cell expansion and inactivate DC cells and CD8^+^ Tc cell activity [79].

Cancer therapies that induce endoplasmic reticulum (ER) stress in cancer cells, such as chemotherapy and photodynamic therapy, ultimately lead to the immunogenic cell death (ICD) of tumor cells [80,81]. ICD is a process in which an adaptive immune response is induced during cell death, thereby establishing a long-term immunological memory. ICD is induced by genotoxic stressors such as chemotherapy, whereby cancer cells undergoing apoptosis release DAMPs to activate ICD in surrounding cells [80]. The type of DAMP released depends on the cell stress response and type of cancer cells, and the elicitation of an anti-tumor response can include the activation of immune cells with anti-tumor activity, inflammasome activation, the initiation of normal antigen presentation and processing, and increased immune-mediated tumor cell killing [82]. While inflammation itself is paramount to cancer regulation, the goal of treatment is to promote tumor-suppressive signals and inhibit or dampen tumor-promoting signals while minimizing chronic inflammation.

### 2.4. The Role of Immune Checkpoint Inhibitors

Immune checkpoints are ligands present on immune cell surfaces that act as negative regulators of immune cell activity to protect the host from autoimmunity. While the signaling mechanisms are not well understood, the ligand expression is regulated by endosomal trafficking [83]. The immune checkpoint blockade mechanism uses monoclonal antibodies, immune checkpoint inhibitors (ICIs), to block the ligand binding between T cells and cancer cells. This interaction reverses immunosuppression, activating and instructing the T cells to destroy cancer cells [84]. Programmed cell death protein 1 (PD-1) on T cells binds to the programmed death ligand 1 (PD-L1) on cancer cells at the tumor site, leading to T cell anergy. In the lymph nodes, cytotoxic T-lymphocyte-associated protein 4 (CTLA-4) on T cells binds to co-receptors B7-1 or B7-2 on antigen-presenting cells (APCs), causing T cell deactivation despite T-cell receptor activation [85,86,87]. The response to immunotherapeutic drugs in cancer is largely attributed to the immunogenicity of the tumor but may also be due to the dose or chemical, biological, or physical barriers that prevent the ICI from engaging in direct interaction [88]. With the discovery of other immune checkpoint ligands and targets, such as the V-domain immunoglobulin suppressor of T-cell activation (VISTA), T cell immunoglobulin and ITIM domain (TIGIT), and T-cell immunoglobulin and mucin domain 3 (Tim-3), new antibodies are being developed and will eventually become available in the pharmaceutical market [89].

The TME landscape is shaped by the activation status and infiltration of CD8+ T cells and NK cells, which, when conditions favor them, can cooperate with their effector functions to combat mutated or malignant cells [90,91]. With the advancement of ICI drugs over the past two decades, recent clinical trials have reported promising results in overall patient responses and survival rates when treated with ICI monotherapy or combination therapy. Anti-PD-L1 and anti-PD-1 antibodies are now applied in the treatment of metastatic melanoma, non-small-cell lung cancer (NSCLC), and renal cell carcinoma (RCC), with average objective response rates of 35–40%, 20%, and 25%, respectively [92].

In breast cancer, TILs are more abundant in the TNBC tumor immune landscape than in non-TNBC tumors, thereby making immunotherapy for TNBC a promising treatment option [93]. Meta-analysis studies reported higher overall response rates when immune checkpoint inhibitors were combined with other conventional drugs. Studies by Zhang and Thomas have illustrated the increased overall survival rates in metastatic TNBC patients receiving adjuvant treatments with pembrolizumab, atezolizumab, or avelumab, with overall response rates ranging from 5.2% to 24% [94,95]. Although ICIs are well tolerated by most patients, the efficacy and treatment outcomes are unpredictable due to the complexity of tumor heterogeneity.

## 3. The Anti-Cancer and Adjunctive Roles of Natural Products in TNBC

**Figure 1:**
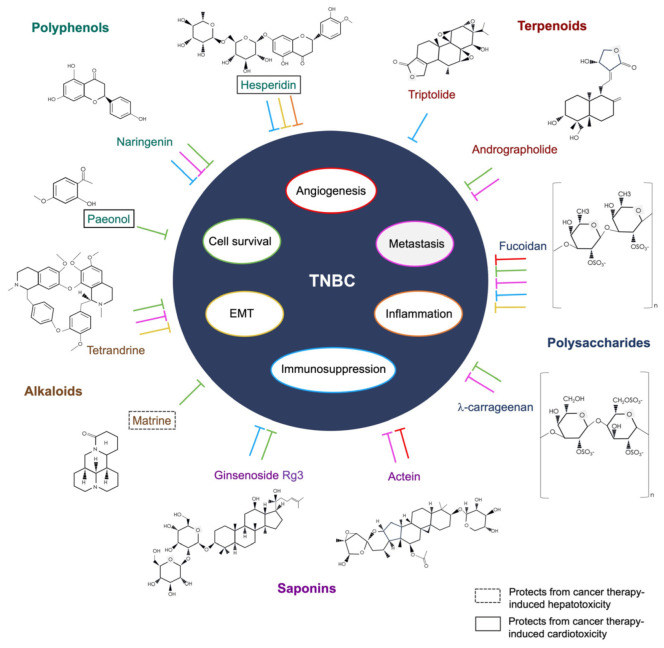
The functional mechanisms of the bioactive compounds on TNBC.

The use of natural products in therapeutics was documented in ancient Egypt and China, based on folk medicine. Medicinal plants and herbs were processed into potions, decoctions, oils, or natural remedies to alleviate complications or treat disease [96]. Isolated morphine and penicillin were among the earliest naturally derived drugs to be commercialized [97]. In later years, the National Cancer Institute organized a large-scale screening program that led to the development of the earliest natural-product-based anti-cancer drugs, taxol, and camptothesin [98]. Despite its success, interest in natural-product-based drug discovery soon waned due to the lengthy and complicated processes involved in acquiring resources, screening, and synthesis [98]. It is estimated that over 60% of all anti-cancer drugs are partially or wholly derived from nature [99]. Currently, natural-product-based drug discovery is focused on a combinational approach, employing the latest technologies and strategies to research molecular interactions, physiology, and therapeutic activities [29]. In this review, the bioactive compounds and their functional mechanisms on TNBC are herein described and summarized in Table 1, and the illustrative representation is summarized in Figure 1.

### 3.1. Polyphenols

The classification of natural products is diverse, but most are derived from plants and fungi as they contain high concentrations of bioactive phytonutrients. Polyphenols can be divided into flavonoids and non-flavonoids, which differ in solubility and chemical composition [138]. Flavonoids are found in the leaves and roots of plants and manifest as a natural protection against plant diseases and parasites, acting as antioxidants and anti-inflammatory agents [139].

Hesperidin, a flavanone glycoside, is abundant in the peel and flesh of citrus fruits, and there is growing evidence that it is beneficial in cardiovascular, neurological, and psychiatric diseases and cancer [140]. Studies suggest that its potent antioxidant effects could not only neutralize free radicals but also provide protection against treatment-induced cardiotoxicity and oxidative stress in tumor-bearing mice, mainly through the production of reactive oxygen species (ROS) [141,142]. Kongtawelert et al. have also shown that hesperidin can downregulate PD-L1 expression in the TNBC MDA-MB-231 cell line [100]. A biocompatible drug delivery model has been developed by Sulaiman and colleagues, whereby gold nanoparticles were bound with hesperidin to treat Ehrlich ascites tumor cell-bearing mice. The results reported a reduction in tumor-induced proinflammatory cytokines IL-6, TNF-α, and IL-1β, and no adverse effects or toxicity in mice has been found [101]. In addition, hesperidin has been reported to reduce the metastatic potential of 4T1 cells aggravated by the chemotherapeutic drug doxorubicin. The reduction of lamellipodia production and downregulation of MMP-9 and Rac-1 protein expression in hesperidin-doxorubicin-treated 4T1 cells suggest possible inhibition or reversal of the epithelial-mesenchymal transition (EMT) in TNBC [102].

Paeonol is a compound found in plants of the *Paeoniaceae* family, and the most relevant species in therapeutic application is *Paenonia suffruiticosa*, commonly known as tree peony. Paeonol possesses anti-anaphylactic, anti-inflammatory, neuroprotective, cardioprotective, and anti-tumor effects [143]. Human MDA-MB-231 cells treated with paeonol have shown reduced proliferation and increased apoptotic activity compared to controls, which may be caused by the interference with the cancer biomarker signaling of C-X-C motif chemokine ligand (CXCL)- 4 and the C-X-C motif chemokine receptor (CXCR)3-B [103,144]. Epirubicin, a potent chemotherapeutic drug used in the treatment of breast cancer, was found to reduce cardiotoxicity and synergistically enhance anti-tumor responses in 4T1 cell tumor-bearing mice when treated with Paeonol [104]. In the context of adjuvant immunotherapy, there are no studies on the use of Paeonol in TNBC and other breast cancer subtypes; however, its adjunctive effects in melanomas have been implicated by targeting the immune checkpoint ligand PD-1 and regulating the microRNA miR-139-5p in thymocytes [145]. The compound is limited by its poor solubility in water and has low bioavailability and high volatility in normal conditions, rendering difficulty in drug delivery without modifications [146].

Naringenin, a flavanone commonly found in citrus fruits and tomatoes, has been implicated in a wide range of biological functions. It has low toxicity and is known to have antioxidant, antidiabetic, hypoallergenic, immunomodulatory, hypolipidemic, and memory-enhancing mechanisms as well as anti-cancer activities [147]. It is known to directly modulate the immune and transcriptional factors involved in acute and chronic inflammatory conditions, including fibrosis, sepsis, diabetes, and cancer [148]. Molecular docking and experiments have confirmed that naringenin could block the phosphorylation of STAT3 and subsequently inhibit the JAK2/STAT3 signaling pathway. In addition, the combination of naringenin and another natural compound, cryptotanshinone, has been shown to enhance the Th1 immune response in mice with spontaneous breast tumors [105,106]. Abaza et al. have shown that naringenin induces cell cycle arrest and apoptosis in breast and colorectal cancer by decreasing cyclin-dependent kinase gene expression and increasing pro-apoptotic genes such as caspases 3, 7, 8, and 9 and Bax [107]. Several studies have demonstrated that naringenin is effective in inhibiting breast-cancer-induced pulmonary metastasis in vivo by reducing the production of TGF-β1 via the PKC signaling pathway [107,108,149]. The immunosuppressive effects of breast cancer were also abrogated by the reduction of infiltrating MDSCs and Treg cells and the upregulation of INF-γ and IL-2-releasing T cells in spleen and lung tissues [108,149].

### 3.2. Alkaloids

Alkaloids are a diverse group of secondary metabolites synthesized from amines in higher plants. They are known to be toxic by nature as a means of protection against herbivorous animals and pests [150,151]. They possess a wide range of therapeutic effects, leading to their application in medicine. Examples include anesthetics and anti-inflammatory and anti-cancer agents such as morphine and quinine [152].

The Chinese medicinal vine plant *Stephania tetranda* S. Moore contains a bisbenzylisoquinoline alkaloid named tetrandrine. Recent clinical studies have reported improved outcomes in patients with COVID-19 and those with silicosis when treated with tetrandrine [153,154]. Aside from its calcium-channel-blocking properties, accumulating evidence suggests that it targets cancer-associated inflammation, induces apoptosis, and reverses multidrug-resistant cancer cell lines [155]. In TNBC, tetrandrine has demonstrated synergetic anti-tumor activities with chemotherapeutic agent trivalent arsenite derivatives in both in vitro and in vivo models of the MDA-MB-231 cell line. The key mediating pathways involved in cancer suppression include the induction of autophagic cell death, S-phase cell cycle arrest, cytotoxic cell death, and the inhibition of metastasis via the PI3K/Akt/mTOR signaling pathway and upregulation of tumor suppressor PTEN [109,110]. Furthermore, tetrandrine contributes to the suppression of EMT and cancer stemness in MDA-MB-231 cells by disrupting superoxide dismutase 1 (SOD1) signaling and increasing reactive oxygen species (ROS) production [111]. ROS serves a dual function in cancer as moderate levels may promote cancer progression and metastasis, but elevated levels could lead to apoptosis. Despite tetrandrine demonstrating many anti-tumor activities via the direct or indirect induction of cancer cell death, its immunological implications in TNBC and other cancers remain to be investigated.

*Sophora flavescenes*, or kushen in Chinese, is a Chinese medicinal herbal root rich in alkaloids and flavonoids. The herb has been used to treat non-communicable and communicable inflammatory diseases, including neurological diseases such as Alzheimer’s disease, cardiovascular diseases, gastrointestinal diseases, and cancer [156,157,158]. One of its bioactive components, matrine, is an alkaloid used in the treatment of various cancers. In addition to targeting apoptosis in cancer cells, other pathways include autophagy-mediated cell death and NK-cell-mediated killing [159]. In vivo application of matrine in a TNBC 4T1-tumor-bearing mouse model has shown significant tumor regression as well as the inhibition of metastasis and angiogenesis [112]. Several studies have demonstrated the autophagic and apoptotic activities induced by matrine in the following human cancer cell lines: Luminal A breast cancer (MCF-7), TNBC and ER-α-negative (MDA-MB-231), ovarian cancer (Hela), and lung cancer (A549) cell lines [113,114]. The PI3K/Akt/mTOR, ERK1/2, and p38 pathways were also activated, indicating that autophagy induction may lead to apoptosis. Although autophagy can promote or suppress tumor growth and its mechanisms are not well defined in cancer, these results were consistent with other cancer studies using matrine, suggesting that autophagy and apoptosis are beneficial against tumor progression and migration [160,161]. The adjuvant use of matrine in clinical trials has been limited as matrine injections have only been tested in small clinical trials in China. The drug has been shown to protect patients with breast cancer from the hepatotoxic effects of chemotherapy [115]. A TCM-based injection, the compound kushen injection (CKI), has been approved for use in China. It consists of aqueous extracts of *Sophora flavescens* and *Smila Glabra*, containing over 200 bioactive compounds, of which the four main compounds are known for their anti-cancer properties: matrine, sophocarpine, oxymatrine, and oxysophocarpine [162,163]. CKI has been used in clinical trials as an adjunctive treatment for cancer patients receiving chemotherapy. In non-small-cell lung cancer (NSCLC), CKI combined with platinum-based chemotherapy improved quality of life, disease control rates, and overall response rates compared with chemotherapy alone [164]. However, results in breast cancer patients were mixed, with some trials reporting no change in clinical responses, while others showed significantly higher clinical responses between the combined treatment group and the group receiving chemotherapy alone [165,166]. Nevertheless, combination treatment resulted in improved quality of life and reduced chemotherapy-related toxicity in these patients, which was consistent across trials.

### 3.3. Terpenoids

Terpenoids are the largest group of secondary metabolites synthesized by plants to deter herbivorous animals and pests but could also serve as attractants to certain insects for pollination. These interactions are mainly mediated by the production of volatile organic compounds from volatile terpenes, which are used in the food and cosmetic industries [167]. Terpenoids are present in marine organisms and plants, providing many benefits to human health through nutritional to therapeutic applications [168,169]. Some known examples of terpenoid-based pharmaceutical drugs include artemisinin and paclitaxel, which respectively treat malaria and cancer [170].

Diterpenes are a subgroup of terpenoids that are most abundant in plants and fungi. Triptolide is derived from the vine *Tripterygium wilfordii*, Hook f., a medicinal plant used mainly in traditional Chinese medicine (TCM). As an immunosuppressant, the drug has been suggested to be beneficial in autoimmune diseases such as rheumatoid arthritis [171]. It has potent anti-inflammatory, cytotoxic, and anti-proliferative properties in several cancers, including TNBC, but the most notable property is its ability to downregulate the immune checkpoint ligand PD-L1 [116]. Triptolide has been shown to potently suppress the expression of PD-L1 in IFN-γ-induced glioma cell lines and oral cancer patient xenograft models [172,173]. This suggests its potential role as an adjuvant to enhance anti-cancer immunotherapy. Despite its potency and efficacy in vitro, it is toxic and poorly absorbed in mammalian models [174]. To address toxicity and drug delivery issues, Luo et al. developed a triptolide-containing thermo-responsive hydrogel for intra-tumoral delivery to 4T1-tumor-bearing mice. The results showed reduced toxicity and increased infiltration of memory T cells [117].

Andrographolide is a diterpenoid lactone isolated from the stem and leaves of the Chinese medicinal herb *Andrographis paniculata* (Burm. f) Wall. ex Nees. This compound has a broad range of pharmacological activities against inflammatory diseases, especially neurological disorders such as Parkinson’s disease, Alzheimer’s disease, multiple sclerosis, and cardiovascular diseases [175,176]. Its anti-cancer properties have been implicated in several cancers, including breast, ovarian, and esophageal cancers [177,178]. In the human breast cancer cell lines MCF-7 and MDA-MB-231, treatment with andrographolide has been shown to reduce proliferation by deactivating the ER-α receptor in MCF-7 cells and activating the apoptosis pathway in MDA-MB-231. This suppresses the downstream PI3K/Akt/mTOR and NF-κB pathways, as well as matrix metalloproteinase (MMP)-9 secretion, resulting in apoptosis and the inhibition of metastasis and angiogenesis [118,119,120]. Also, andrographolide has been shown to sensitize TNBC cells to doxorubicin via the downregulation of IL-6-mediated STAT3 phosphorylation, resulting in enhanced cancer cell cytotoxicity [121].

### 3.4. Bioactive Polysaccharides

Polysaccharides are macromolecules composed of glycoside-linked monosaccharide subunits found in most plants, fungi, bacteria, and marine algae. Bioactive polysaccharides are synthesized and utilized by living organisms for biological functions and have therapeutic or pathogenic applications [179]. These bioactive compounds are generally non-toxic and offer many benefits to human health, in particular, the regulation of the gastrointestinal tract, which facilitates the production of fermentation by-products and the dilution of toxins. This is important in the prevention of chronic inflammatory diseases and immune dysfunction [180,181].

*Cladosiphon okamuranus* is a type of brown algae seaweed found in East Asia and is part of the Asian diet as a rich source of fiber. It contains fucoidan, a sulfated polysaccharide known for its anti-cancer and antioxidant activities against various solid cancers [182]. Some of its effector functions in cancer include the induction of apoptosis and suppression of angiogenesis and metastasis. This was demonstrated by the inhibition of the Akt/MAPK/PI3K signaling pathway and its downstream transcription factors, NF-κB and AP-1, in the human TNBC MDA-MB-231 cell line [122]. Another study has reported the arrest or reversal of the EMT in the MDA-MB-231 and 4T1 cell lines, degradation of the transforming growth factor receptor II (TGFRII) in MDA-MB-231 cells, and suppression of lung metastasis in 4T1 cell line tumor-bearing mice [123]. Fucoidan nanoparticles and oral supplements have been developed to enhance drug delivery for both in vitro and in vivo models in its adjuvant application. Fucoidan containing a cationic polyethyleneimine structure carrying doxorubicin has been demonstrated to enhance the cytotoxic effects of doxorubicin in 4T1 cell tumor-bearing mice, which is regulated by the manipulation of the tumor immune landscape, shifting from M2 to M1 TAM phenotype polarization and increased Th1 immune response [124]. Another study used oligo-fucoidan aqueous extract supplements, a low-molecular-weight Fucoidan, and illustrated the enhanced effect of the chemotherapeutic drug olaparib on a 4T1 tumor cell line tumor-bearing mouse model by suppressing the production of inflammatory IL-6, phosphorylated epidermal growth factor receptor (p-EFGR), and PD-L1. Fucoidan also attenuated the immunosuppressive effects of olaparib by reducing the Treg cell population and M2 macrophages in tumors, leading to increased tumor immunogenicity and preventing cancer recurrence in mice after surgery [125].

Carrageenan oligomers are oligosaccharides derived from rodophyceae or red algae seaweed. There are three main types of carrageenan oligomers: kappa(κ), iota(ɩ), and lambda (λ), with λ-carrageenan oligosaccharides having the highest degree of sulfation [183,184]. These oligosaccharides are commonly used as texture enhancers in the food industry because of their gel formation abilities but are also relevant in neutraceutical research as they possess antiglycemic, immunomodulatory, and prebiotic properties [185]. Studies in cancer have shown that λ-carrageenan suppresses metastasis and heparinase activities and induces apoptosis in the human MDA-MB-231 cell line, actively inhibiting tumor invasion [126,127,128]. λ-Carrageenan when administered by intratumoral injection has been demonstrated to significantly reduce tumor growth and promote the infiltration of CD11c^+^ dendritic cells and F4/80^low^ M1 macrophages into the tumor. It also enhanced anti-cancer immune responses when treated as an adjuvant to an OVA-based prophylactic cancer vaccine in both B16-F10 melanoma mouse models and 4T1 mouse TNBC models [129].

### 3.5. Saponins

Saponins occur naturally in the form of triterpenoid glycosides or steroids and play a role in plant defense systems against fungal and bacterial infections and mollusk infestations [186]. There are many structural variations of saponins, but one common feature is their amphipathic nature that allows for foam formation [187]. It is, therefore, widely used in the chemical, cosmetic, agricultural, and pharmaceutical industries. Their biological functions include complex formation with cholesterol, permeabilization of cell membranes, the induction of cell death, and the regulation of signaling processes [188].

The renowned medicinal root used in East Asian traditional herbal medicine, *Panax ginseng* C.A. Meyer, more commonly referred to as Korean ginseng, has been used to treat common ailments such as fatigue and physical weakness [189]. *Panax ginseng* contains the bioactive component ginsenosides, a group of saponins with immunostimulatory, neuroprotective, and anti-inflammatory activities. Ginsenoside Rg3, which is abundant in the roots of Korean red ginseng, has been well-studied in the literature for both chemopreventive and anti-cancer activities [190,191]. It has been shown to inhibit the NF-κB signaling pathway and induce mitochondrial-mediated apoptosis and cell cycle arrest in breast cancer cell lines MDA-MB-231 and 4T1 [130,131]. Rg3 can perform dual functions when human TNBC cells were treated in combination with the chemotherapeutic drug paclitaxel. Rg3 demonstrated the enhancement of paclitaxel-induced apoptosis in MDA-MB-231, MDA-MB-453, and BT-549 cell lines. Also, Rg3 reversed chemoresistance in the modified MDA-MB-231 cell line (MB231-PR cells) with paclitaxel resistance [132,133,134]. Regarding the chemical properties of ginsenosides, they are insoluble in water and poorly soluble in the gut. Nevertheless, modern techniques in drug delivery systems have been developed to increase permeability and targeting, thus potentiating its application as an adjunctive treatment for cancer [192]. For example, liposome or nanoparticle packaging for ginsenosides is currently being developed to ensure that bioavailability, efficiency, and toxicity are not compromised [193,194]. Studies on the application of ginsenosides in chemoimmunotherapy and nanotechnology are increasing, whereby ginsenoside-chitosan hydrogel-encapsulated doxorubicin could enhance ICD effects on 4T1-tumor-bearing mice and increased memory T cell infiltration into the TME [135].

The genus *Cimicifuga*, which includes *Cimicifuga foetida*, *Cimicifuga dahurica*, and *Cimicifuga racemosa* (L) Nutt. are medicinal plants used in East Asia and North America to relieve inflammatory conditions such as sore throats, viral infections, and complications of gynecological disorders [195,196]. The bioactive triterpenoid glycoside, actein, found in the rhizomes of *Cimicifuga* plants, has been shown to exert anti-cancer and anti-HIV activities [197]. In TNBC, Wu et al. and Yue et al. have demonstrated that actein can potently inhibit metastasis in an MDA-MB-231 human breast cancer zebrafish xenograft model and 4T1 tumor mouse model. Other effector functions of actein include the deactivation of the Akt, NFkB, and JNK/ERK signaling pathways and the downregulation of EGFR expression in the MDA-MB-231 cell line [136,137]. In a recent study, new variants and derivatives of actein have been successfully synthesized by its incorporation into pharmacophores, resulting in increased potency and the better delivery of anti-proliferative activities in human TNBC cell lines compared to actein itself [198]. These findings suggest that the novel compound and its derivatives may be promising candidates for adjuvant cancer therapy, although research on its synergistic effects and immunomodulatory mechanisms is lacking.

## 4. Discussion

The potential benefits of natural products are not limited to adjunctive therapeutics but also have nutraceutical and pharmacological applications, depending on their efficacy and toxicity profiles. Although there is a lack of clinical trials on the use of natural products as adjuvants to immunotherapy, also known as phyto-immunotherapy, it is a promising area of research for the discovery of alternative methods to improve cancer therapy [199]. Studies and clinical trials of natural-product-based chemotherapy are gaining traction, potentially offering a safer and more effective approach to treatments.

The main pathways in which natural products are involved against inflammatory diseases include the induction of programmed cell death, suppression of chronic inflammation, and modulation of gut immunity [200]. The gut microbiota is an essential part of immune regulation, with natural-product-derived drugs and supplements playing significant roles in shaping the microbiome. This also ensures that cancer-treatment-induced dysbiosis can be restored by enriching beneficial bacteria and depleting harmful ones [201]. Chemotherapy drugs can interfere with normal gastrointestinal functions and reduce healthy gut microbiota, which may compromise the overall treatment efficacy or promote drug resistance [202]. From the perspective of Western medicine, manipulation of the gut microbiota is achieved through direct interventions, including probiotics, neutraceuticals, antibiotics, and fecal transplants [203]. In contrast, TCM herbal formulas are based on holistic treatments that use crude herbal extracts to influence systemic immunity. In adjunctive cancer treatment, TCM could alleviate conventional-treatment-induced toxicity and improve the well-being of patients. However, while they are crude extracts rich in phytonutrients, their mechanisms of action are imprecise and difficult to elucidate, and the methods are limited to a holistic level [204]. As a result, drug discovery, whether from natural or synthetic sources, is heavily reliant on mechanistic studies to research targeted treatments. The study of adjunctive phytoimmunotherapy to investigate synergistic anticancer and immunomodulatory activities may forge a connection between these distinctive treatment approaches.

A major obstacle to the application of bioactive compounds is ensuring specificity in targeting tumor-mediated inflammation. Since cancer is a disease of chronic inflammation, and inflammation is highly context-dependent in terms of net pro- or anti-tumorigenic effects, natural products, despite their anti-inflammatory potential, may not be sufficient to offset the pro-tumorigenic setting in the TME. Bioactive compounds from natural sources are promising adjunctive candidates to provide immunological support to TNBC patients undergoing immunotherapy. It is noteworthy that despite the observation of positive results from natural products, most compounds would not be translatable into clinical trials. The pharmacokinetics, stereochemistry, and bioavailability of the compounds are of fundamental importance for successful drug delivery as the chemical composition and shape of the bioactive molecule can influence downstream signaling mechanisms [205]. Also, the same natural products purified and obtained from different plant species or plant structures are likely to differ significantly in bioactivity and potency.

If the natural bioactive compounds are approved and commercialized, there will be major drawbacks in large-scale cultivation and production to fulfill demands, one of which is the potential environmental implications of disrupting the biological diversity of the sources as the acquisition, isolation, and synthesis of some compounds require large quantities of resources [206]. Therefore, it is essential to ensure a stable resource supply with minimal environmental impact, should the product be popularized. The notion that TNBC tumors may exhibit intertumoral and intratumoral heterogeneity, particularly of different subtypes, raises questions about the efficacy of natural products, despite their ability to target multiple pathways simultaneously. For example, they may be effective in some subtypes but resistant in other subtypes within the same tumor. Another concern over adjunctive immunotherapy is treatment durability. Since chronic and acute inflammation often occurs simultaneously during immunogenic cell stress, the downstream signaling effects can lead to three outcomes: tolerogenic cell death, immunomodulation, or ICD [207]. The induction of ICD is a reliable indicator of treatment success and tumor elimination as it provides a long-term immunological memory to prevent future cancer relapse. All three processes are required for the successful execution of ICD: the activation of premortem stress and programmed cell death, expression of ICD-related immunomodulatory biomarkers, and immunomodulatory biomarker-mediated activation of acquired immunity [208]. It is uncertain whether immunological stimulation by natural compounds is sufficient to activate ICD and provide consistent and favorable outcomes.

Future technology may incorporate natural products into better drug delivery systems, such as the use of nanoparticles and controlled dosing modalities. Molecular docking and pharmacological networking could be used to facilitate the screening and biocompatibility testing of potential compound candidates. A growing number of studies have successfully applied and tested natural products with engineered biomaterials, which have made outstanding contributions to pharmaceutical research. As modern integrative medicine evolves and influences treatment efficacy, modulating gut microbiota, systemic immunity, and TME is a key target for successful cancer treatment.

## 5. Conclusions and Future Directions

In conclusion, there are many advantages of using natural products to sensitize immunotherapeutic drugs in combating TNBC and improving the overall quality of life. Cancer is a complex disease with an unpredictable and ever-changing microenvironment, with the capability to adapt to substances. However, in the era of technological advancements, new research platforms can be developed to investigate pharmaceutical profiles. In our current understanding of tumor immunology, molecular signatures differ between tumors and individuals, and the latest treatment options are not tailored to each patient. It is, therefore, imperative to develop safe, effective, and versatile therapies that provide long-term benefits to systemic immune regulation to minimize the risk of cancer recurrence and improve health outcomes. Novel interventions and modalities are constantly being developed. It is proposed that future cancer therapies will include testing immunotherapy drugs in combination with a cocktail of various natural products. This not only provides insights into drug interactions and mechanisms but also offers the possibility of discovering novel bioactive compounds from unconventional sources, such as marine organisms, insects, and bacteria. Currently, immunotherapy alone is insufficient to reduce the tumor burden or provide long-term immunological benefits in most TNBC patients.

## Figures and Tables

**Table 1:** Summary of natural product derivatives and their pharmaceutical activities on TNBC.

Class	Compound	Biological Functions in TNBC	Ref.
Polyphenol	hesperidin	Downregulation of PD-L1 expression in TNBC MDA-MB-231 cells	[100]
		Downregulation of IL-6, TNF-α, and IL-1β in Ehrlich ascites tumor-bearing mice	[101]
		Suppression of EMT in 4T1-tumor-bearing mice	[102]
	paeonol	Inhibition of proliferation and increased apoptosis in MDA-MB-231 cells	[103]
		Suppression of Epirubin-induced cardiotoxicity in 4T1 cell tumor-bearing mice	[104]
	naringenin	Enhancement of cryptotanshinone- or cyclophosphamide-induced cytotoxicity and suppression of metastasis in spontaneous mammary tumor-bearing mice	[105,106]
		Suppression of proliferation and enhancement of immunogenicity	[107,108]
Alkaloid	tetrandrine	Enhanced autophagic cell death and S-phase cell cycle arrest in MDA-MB-231 cell line and mouse xenograft models	[109]
		Cytotoxic cell death and inhibition of metastasis in MDA-MB-231 cell line	[110]
		Inhibition of EMT and cancer stemness in MDA-MB-231 cell line	[111]
	matrine	Inhibition of metastasis and angiogenesis in 4T1 cell-tumor-bearing mice	[112]
		Promotion of autophagy and apoptosis in MDA-MB-231, Hela, and A549 cells	[113,114]
		Suppression of chemotherapy-induced hepatotoxicity in breast cancer patients	[115]
Terpenoid	triptolide	Downregulation of PD-L1 expression in MDA-MB-231 cells	[116]
		Enhancement of immunogenicity in 4T1-tumor-bearing mice	[117]
	andrographolide	Promotion of apoptosis and inhibition of metastasis and angiogenesis of MCF-7 and MDA-MB-231 cells	[118,119,120]
		Sensitization to doxorubicin-induced cytotoxicity in MDA-MB-231 cells	[121]
Bioactive Polysaccharide	fucoidan	Promotion of apoptosis and inhibition of metastasis and angiogenesis in MDA-MB-231 cells and inhibition of EMT	[122,123]
		Enhanced immunogenicity	[124]
		Downregulation of PD-L1 expression and reversal of Olaparib-induced immunosuppression in 4T1 cell-tumor-bearing mice	[125]
	lambda (λ)-_carrageenan	Inhibition of metastasis and tumor invasion and induction of apoptosis in MDA-MB-231 cells	[126,127,128]
		Enhancement of immunogenicity and anti-cancer immune responses in 4T1-tumor-bearing mice	[129]
Saponin	ginsenoside R3	Promotion of apoptosis and cell cycle arrest in MDA-MB-231 and 4T1 cells	[130,131]
		Enhancement of paclitaxel-induced cytotoxicity and reversal of paclitaxel resistance in human TNBC cell lines	[132,133,134]
		Enhancement of immunogenicity and doxorubicin-induced immunogenic cell death in 4T1-tumor-bearing mice	[135]
	actein	Inhibition of metastasis and angiogenesis	[136,137]
		Inhibition of proliferation by inducing cell cycle arrest	

## Data Availability

Not applicable.

## Sample Availability

Not available.
